# 

**Published:** 1910-01-01

**Authors:** 


### BOOKS RECEIVED.

Reynolds-Balls.
"Health Resorts of Europe." By Thomas Linn, M.D.

Scientific Press, Ltd.
"An A B C of Nursing in Accidents and Illnesses." By E. M. Clarke, C.O.M.

Sampson Low, Marston and Co., Ltd.
"A Beautiful Arm." By Amos Booth.

John Bale, Sons and Danielsson, Ltd.
"The Diet Factor in Disease." By George Black, M.B.

Eveleigh Nash.
"Home Exercises for Health and Strength." By Filip Sylvan, M.D.

Henry Frowde and Hodder and Stoughton.

"A System of Operative Surgery, Vol. I., III. By F. F. Burghard, M.S., F.R.C.S.

### Gifts and Bequests.

The Girdlers' Company has distributed between £400 and £500 among certain hospitals and charitable institutions in and about London.

Mrs. Hannah Botterill has bequeathed £50 each to the Hull Royal Infirmary and the Scarborough Royal Hospital and Dispensary.

The treasurer of the Bristol General Hospital acknowledges the receipt of a donation of £250 from the chairman (Mr. Herbert M. Baker) towards reducing the debt of over £5,000. Amounts received: Anonymous, £1,000; Mr. Joseph Storrs Fry, president, £1,000.

Lord Sandhurst, treasurer of St. Bartholomew's Hospital, acknowledges receipt of the following further donations in response to the appeal by the Lord Mayor in October:—Messrs. J. H. Schröder and Co., £500; Lloyds Bank, Limited, £105; "R. E. J.," £50; and Messrs. Stephenson, Clarke, and Co., £26 5s.

Mrs. Esther Israel has left £50 to the Jews' Orphan Asylum and Hospital at Norwood, the income to be divided annually between two boys who shall say Kadish to her late husband (Kadish being the Jewish form of prayer and thanksgiving at funerals) on the anniversary of his death; the sum of 25 guineas each to the Charing Cross Hospital, King's College Hospital, the Jewish Home for Incurables, Tottenham, the London Hospital, and the Jews' Hospital and Orphan Asylum. Subject to certain trusts, she left £1,000 to the London Hospital for the endowment of a bed, and £200 to the Charing Cross Hospital for general purposes.

Additional contributions in response to the appeal on behalf of the special fund for the liquidation of the capital debt of St. Bartholomew's Hospital are as follows:—Messrs. N. M. Rothschild & Sons and Mr. Otto Beit, £500 each; Sun Insurance Office, £200; Messrs. Antony Gibbs and Sons, the Union of London and Smiths Bank, Limited, Lord Revelstoke, and Sir Alexander Henderson, Bart., £105 each; Messrs. Glyn, Mills, Currie, and Co., £100; Messrs. Cook, Son and Co. and Mr. E. B. I'Anson, £52 10s. each; Mr. Samuel Figgis, £50; Mr. Edwin Waterhouse, £26 5s.; Mrs. Henry L. Cohen, £25; and Speyer, Schwerdt and Co., Limited, £21. The total amount received so far is £11,665, and further assistance is urgently needed.

The Fishmongers' Company has made the following grants: Royal Free Hospital School of Medicine for Women, £25; All Saints' Convalescent Hospital, Eastbourne (for Christmas entertainment), £3 3s.; Billingsgate Mission and Dispensary, £10 10s.; Brompton Hospital for Consumption, £31 10s.; City of London Truss Society, £21; General Lying-in Hospital, York Road, S.E., £3 3s.; London Hospital, £52 10s.; Mount Vernon Hospital for Consumption, Hampstead, £18 18s.; National Hospital for Diseases of the Heart, Soho Square, £6 6s.; National Truss Society, King William Street, E.C., £10 10s.; Queen Charlotte's Lying-in Hospital and Royal Dental Hospital, each £5 5s.; Royal Free Hospital, £21; Royal General Dispensary, Bartholomew Close, £10 10s.; Royal Maternity Charity, £5 5s.

### Appointments.

The medical officer at Oswestry, Dr. R. de la Poer Beresford, has resigned in order to become Mayor.

Mr. T. Bates, jun., F.R.C.S., has been appointed honorary surgeon to the Worcester General Infirmary.

Mr. Graham Simpson, F.R.C.S., has been appointed Lecturer in Operative Surgery at the University of Sheffield.

Mr. Chas. Holman has been temporarily appointed to the position of medical officer to the Uckfield Board of Guardians.

Gerald S. Hughes, M.B. B.S.Lond., F.R.C.S.Eng., has been appointed surgeon (with care of out-patients) to the York County Hospital.

The Hanley Education Committee has appointed Dr. Dorothy W. Stevenson, clinical assistant, Edinburgh Royal Infirmary, school medical officer.

Dr. C. R. Salisbury, assistant medical officer in the education department of the London County Council, has been appointed medical officer at the Hanwell Institution of the Central London School District.

Dr. Ernest Watt, senior assistant to the medical officer for the county of Lanark, has been appointed medical officer of health, superintendent of Knightswood Hospital, and police surgeon for the burgh of Partick.

Dr. Henri de Rothschild's latest play, "La Rampe," remarks the *Chemist and Druggist*, has had a successful run at the Gymnase Theatre. The final and most striking incident is the suicide of the heroine. Poisoning scenes in dramas and novels are often notoriously untrue to nature; but the fact that this piece is written by a practising M.D. is a guarantee to the spectators that the effects of a *petit verre* of aconite are accurately depicted by Mlle. Brandès, although it is possible that the average lady actress dies on the stage in a less graceful and affecting attitude.

### THE GRADUATES' MEDICAL SCHOOLS.

DIARY FOR THE WEEK, JANUARY 3 to 7.

### ST. JOHN'S HOSPITAL FOR DISEASES OF THE SKIN, Leicester Square, W.C.

Chesterfield Lectures.—At the Hospital on Thursday evenings at 6 p.m., each lecture followed by clinical demonstrations :—

**1910—Jan. 6**, Essentials in the Study of Dermatology.

### MEDICAL GRADUATES' COLLEGE AND POLYCLINIC, 22 Chenies Street, W.C.

**At 4 p.m.—Jan. 3**, Dr. James Galloway, **Skin.**
**At 5.15 p.m.—Jan. 3**, Dr. J. E. R. McDonagh, **Serum Diagnosis of Syphilis.**
**At 4 p.m.—Jan. 4**, Dr. H. Campbell, **Medical.**
**At 5.15 p.m.—Jan. 4**, Mr. H. Stansfield Collier, **Indications for Abdominal Operations.**
**At 4 p.m.—Jan. 5**, Mr. Edred M. Corner, **Surgical.**
**At 5.15 p.m.—Jan. 5**, Dr. James Donelan, **Suppuration in the Maxillary Antrum.**
**At 4 p.m.—Jan. 6**, Sir Jonathan Hutchinson, **Surgical.**
**At 5.15 p.m.—Jan. 6**, Dr. George Oliver (Harrogate), **The Treatment of High Blood Pressure.**
**At 4 p.m.—Jan. 7**, Mr. J. Gay French, **Ear, Nose, and Throat.**

### CENTRAL LONDON THROAT AND EAR HOSPITAL, Gray's Inn Road, W.C.

**At 4.30 p.m.—Jan. 6**, Dr. Dundas Grant, **Laryngeal Tuberculosis.**

### ROYAL SOCIETY OF MEDICINE, 20 Hanover Square, W.

**At 8.30 p.m.—Jan. 4. Pathological Section:**
Laboratory Meeting at University College Hospital Medical School (Entrance in University Street).

*Communications :*

Mr. W. Trotter : "Peritoneal pseudo-myxoma arising from the appendix."
Mr. T. Lewis : "Auricular fibrillation."
Mr. E. H. Starling and Mr. C. Bolton : "On the blood-pressures and lymph flow in a case of heart disease in the dog."
Mr. F. H. Thiele : "Observations on the metabolism of fat."
Mr. D. N. Nabarro : "Acid-fast bacilli in butter."
Mr. C. Bolton : "Effects of motor insufficiency of the stomach upon the healing of acute gastric ulcer."
Mr. T. W. P. Lawrence : "Total necrosis of the kidney."

*Demonstrations :*

Mr. C. Russ : Electrical reactions of certain bacteria.
Mr. A. Payne : Specimens of mouse cancer.
Mr. J. H. Parsons and Mr. E. E. Henderson : Effects of ultra-violet light on the lens capsule.
Mr. H. B. Shaw : Tuberculous pericarditis.
Mr. G. S. Hett : An unusual case of human glanders.
Mr. A. C. Stevenson : Intestinal coccidiosis in the goat (Khartum).

**At 5 p.m.—Jan. 7, Laryngological Section:** Cases and Specimens.

Dr. Ham, chairman of the Victorian Board of Public Health, thinks that cinematograph views illustrating technical and educational subjects should be shown in the schools as an aid in the instruction of the pupils. Dr. Ham states that Edison expresses his entire concurrence with the view, and points out that in France something of the kind is done. For the purpose of lecture work Dr. Ham is now importing a set of films dealing with surgical and medical subjects. These will show the typhoid and other organisms at work, as revealed by the microscope.

### THE QUESTION BOX.

**SPECIAL NOTICE.**—Questions of interest affecting the honorary medical staff and hospital administration in all departments are invited. When any point arises we hope our readers will formulate it in a question and so co-operate to make this column of real value and interest. In this way the experience of the best-managed hospitals should be made helpful to the whole hospital world. We shall welcome the contribution of both questions and answers.

N.B.—The publication of an answer in this column does not necessarily preclude the publication of subsequent answers to the same question, for a question may involve points which require to be threshed out and fully discussed. Answers, if published, will be paid for at scale rates.

### THE COMMITTEE OF MANAGEMENT AND THE HONORARY MEDICAL STAFF.

### THE QUESTION OF REPRESENTATION.

Question 1: Is it desirable that the Honorary Medical Staff should have seats on the General Committee, or that they should meet as a Medical Board and recommend to the General Committee any change they may consider necessary?

#### Manchester Views.

By Mr. W. G. Carnt, General Superintendent and Secretary, Manchester Royal Infirmary.

I am strongly of opinion that to administer a hospital effectively and without friction, it is essential that the honorary medical staff should be represented upon the Committee of Management. The General Committee consists of a number of gentlemen, elected by the governors to administer the hospital, because of their known business capabilities, their position in the town or county in which the hospital is situated, the interest they have displayed in the welfare of the institution, or the financial assistance they have rendered it. In many instances the members possess the whole of these qualifications, and the institution enjoys the benefit of their business acumen, the prestige of their association with its administration, and their substantial pecuniary assistance in times of financial anxiety. The General Committee do not, as a rule, however, possess medical knowledge, and, however well qualified they may be to conduct the important business relations of the institution, questions inevitably arise during their deliberations upon which a medical opinion is of the utmost value, and it simplifies matters considerably to be able to ask a medical member a question, get an immediate answer, and dispose of the business forthwith. The presence of representatives of the honorary medical staff upon the General Committee saves many references of semi-important matters to the whole staff, a process which causes delay and may possibly occasion misunderstanding, and, moreover, when an important matter arises upon which the lay committee desire to have the opinion of the whole honorary staff, it is of material assistance for some of the staff to be acquainted with the reasons for the reference and the feeling of the lay committee with regard to it, the subject is approached from a proper standpoint, and the risk of misunderstanding is reduced to a minimum. There are also other distinct advantages. These medical representatives learn a good deal about the difficult question of finance. They see the close examination of expenditure which is characteristic of the best hospital committees, the invariable insufficiency of income, and the difficulty of making both ends meet, and they carry away with them to meetings of the Medical Board and to their work in the hospital a spirit of economy which cannot but assist in keeping the expenditure of the charity as low as efficiency permits. They witness also the methods of administration, and have an opportunity of realising the spirit with which general committees approach the many serious problems with which they have to deal. They see for themselves the keen business ability, sound judgment, and absolute fairness which, during a long experience, I have always found prevailing in the board room of a voluntary hospital, and they can realise, if they consider fairly and dispassionately the proceedings of the General Committee, that if, in the interests of the governors and subscribers, or in those of the sick poor for whose benefit the charity exists, they have to refuse a request from the honorary medical staff, dissent from a recommendation, or express a desire for amendment in any particular, it is done with the intention of benefiting the hospital and with no desire to wound the susceptibilities of the medical staff. If the staff are not represented upon the committee of management, practically the only method of communication between the two bodies is by resolutions sent in writing the one to the other. When these are produced there is no one present able to explain what was in the minds of the framers, and the too frequent consequent result is that misunderstandings arise and friction is rampant.

The reply to Question No. 1 is, therefore, that the medical staff should be represented upon the General Committee, and should also from time to time meet as a medical board to receive reports from their representatives and to instruct them as to their future policy, to consider matters of moment referred to them by the General Committee, or to formulate recommendations upon matters of such importance that a representation from the entire medical board is considered necessary.

By "Hospital Secretary."

With reference to the question of the honorary medical and surgical staff being represented upon the General Committee, I would submit that it is certainly most desirable in the interests of a voluntary hospital that the members of the honorary staff should have seats on the governing body.

#### A Newcastle Practice.

At the Royal Victoria Infirmary, Newcastle-on-Tyne, the whole medical and surgical staff meet as a committee and forward reports to the House (Management) Committee, either on their own initiative or on matters which have been referred to them for consideration by the House (Management) Committee.

#### A Birmingham View.

By Mr. Howard J. Collins, House Governor, Birmingham General Hospital.

Yes; both. The senior honorary staff should be on the Managing Committee, and the whole of the visiting staff (senior and junior physicians and surgeons and specialists) should form a medical committee and have regular meetings and make recommendations to the committee or board of management.

### A CHRISTMAS APPEAL FOR SAILORS' ORPHANS.

Twenty-seven thousand and forty-six sailors of the British merchant navy have lost their lives by shipwreck and casualties to ships at sea during the past twenty-five years. A low estimate places the number of their orphan children at 45,000, of whom a very large proportion were left destitute. During the same period the Royal Merchant Seamen's Orphanage has been able to provide for only 1,333, and an earnest appeal for help is made to all who appreciate the hardships, the heroism, and the perils of a sailor's calling, and its importance to the nation. The smallest gift will be thankfully acknowledged by the Secretary, at Dixon House, Lloyd's Avenue, London, E.C. Bankers: London County and Westminster Bank.

